# Tick-borne encephalitis virus subtypes: mono- and mixed infection in specific and non-specific ticks

**DOI:** 10.3389/fcimb.2025.1568449

**Published:** 2025-03-26

**Authors:** Alexandra E. Polienko, Oxana A. Belova, Alexander G. Litov, Anastasia A. Rogova, Galina G. Karganova

**Affiliations:** ^1^ Laboratory of Biology of Arboviruses, Chumakov Federal Scientific Center for Research and Development of Immune-and-Biological Products of Russian Academy of Sciences (Institute of Poliomyelitis), Moscow, Russia; ^2^ Department of Organization and Technology of Production of Immunobiological Preparations, Institute for Translational Medicine and Biotechnology, First Moscow State Medical University (Sechenov University), Moscow, Russia

**Keywords:** tick-borne encephalitis virus, subtype, *Ixodes*, *Dermacentor reticulatus*, mixed infection, infectivity, reproduction level

## Abstract

Tick-borne encephalitis virus (TBEV) is traditionally divided into three main subtypes – European (Eu), Siberian (Sib) and Far Eastern (FE), the distribution of which is confined to the areas of the main vectors, *Ixodes ricinus* (TBEV-Eu) and *Ixodes persulcatus* (TBEV-Sib, TBEV-FE). *Dermacentor reticulatus* also can act as competent vector and participate in TBEV circulation together with the main vectors. It is suggested that there is a specific adaptation not only between TBEV variant and certain tick species, but also between virus variant and local populations of one tick species. In our study, we percoxally infected two populations of *I. ricinus, I. persulcatus* and *D. reticulatus* collected in remote areas with three TBEV strains of the main subtypes. Dynamics of the number of TBEV RNA copies and of the number of infectious for mammalian cells virus particles during mono- and mixed infection of ticks were estimated by real-time PCR and plaque assay in PEK cell culture, respectively. Data was obtained that *I. ricinus, I. persulcatus* and *D. reticulatus* effectively support the reproduction of TBEV regardless of the strain. Interpopulation differences of local populations of one tick species in the maintenance of TBEV reproduction were revealed in *I. persulcatus* during mono- and mixed infection and in *I. ricinus* during mixed infection. Despite minor differences in the level of virus reproduction in ticks, we observed changes in the infectivity of TBEV strains for mammalian cell culture during persistence in different species of ticks. Notably, the TBEV-Eu increased infectivity during adaptation to a non-specific tick species. Thus, we demonstrated that the level of virus reproduction is not the primary factor that determines the adaptation of TBEV to a new tick species. The nature of changes in TBEV infectivity depends on the virus strain and the species of ticks.

## 1 Introduction

Tick-borne encephalitis virus (TBEV) belongs to the genus *Orthoflavivirus* (*Flaviviridae*). Its genome consists of a single-stranded positive-sense RNA with a single open reading frame (ORF), flanked by 5’ and 3’ untranslated regions. The ORF, 11,000 nt, encodes a polyprotein that is co- and post-translationally cleaved into 3 structural proteins (C, prM, E) and 7 non-structural proteins (NS1, NS2A, NS2B, NS3, NS4A, NS4B, NS5) (Růžek et al., 2019).

The initial classification based on antigenic properties and nucleotide sequence of the E protein divided TBEV into three subtypes: European (TBEV-Eu), Siberian (TBEV-Sib), and Far-Eastern (TBEV-FE) (Ecker et al., 1999). This classification closely matched the geographical distribution of the subtypes. Currently, a genotype-based classification has been proposed, with a 10% or more nucleotide sequence difference separating genotypes. This has resulted in seven TBEV genotypes: the three main subtypes (TBEV-Eu, TBEV-Sib, TBEV-FE), genotype 4 (strain 178-79), genotype 5 (Baikalian subtype), Himalayan, and Obskaya genotypes (Deviatkin et al., 2020). Some authors consider the Obskaya genotype as a Siberian subtype lineage (Tkachev et al., 2020). For TBEV-Eu, TBEV-Sib, and TBEV-FE, antigenic differences have been observed, and the identification of new genotypes is based solely on phylogenetic analysis, with no explanations available for such divisions, aside from geographic isolation (Demina et al., 2012; Tkachev et al., 2017a; Dai et al., 2018; Adelshin et al., 2019).

Over the past decade, the overall number of TBEV cases in Russia has decreased (1.9 per 100,000 population in 2012, 1.34 per 100,000 population in 2022) (Nikitin et al., 2024). Meanwhile, in Europe, the number of registered TBEV cases has increased in the last decade (Tick-Borne Encephalitis, 2022). At least one-third of acute forms result in a long period of convalescence and disability. Studies suggest that 40–50% patients with TBE develop a post-encephalitic syndrome (Růžek et al., 2019), and the development of a chronic infection with subsequent reactivation of TBEV is possible (Volok et al., 2022). It was previously believed that subtypes differed in their clinical manifestations. TBEV-Eu is characterized by a low mortality rate (1-2%), a biphasic disease course, predominance of mild forms, and absence of severe neurological outcomes (Zavadska et al., 2018; Hellenbrand et al., 2019); TBEV-Sib has a mortality rate of 6-8%, with chronic cases reported (Gritsun et al., 2003b, Gritsun et al., 2003a; Poponnikova, 2006); TBEV-FE is always associated with the most severe forms of the disease and a mortality rate of 20-40% (Leonova et al., 2013). This subtype characterization does not take into account many social factors, and for each subtype, a variety of clinical manifestations have been described, ranging from asymptomatic cases to severe encephalomyelitis forms (Leonova et al., 2013; Bogovič et al., 2022).

TBEV spread is closely linked to the distribution of ticks, its main vectors: TBEV-Eu is associated with *Ixodes ricinus* and is widely distributed across Europe; TBEV-Sib is linked to *Ixodes persulcatus* and is found in Siberia, the Russian Far East, and northern Europe, including the Baltic countries and Finland. TBEV-FE circulates in the Russian Far East and is also associated with *I. persulcatus*. The initial association of TBEV subtypes with specific vectors does not explain the division of the virus into subtypes, since *I. persulcatus* is the main vector of both TBEV-Sib and TBEV-FE.

Currently, the geographic range of TBEV has shifted, and subtypes are being detected in new territories: TBEV-Eu has been found in Korea, while TBEV-FE was identified in China and Japan (Zhang et al., 2012, Zhang et al., 2016; Yoshii et al., 2017). TBEV-Sib has been detected in northwestern Russia (Republic of Karelia) and neighboring northern European countries (Bugmyrin et al., 2013; Katargina et al., 2013; Laaksonen et al., 2017). TBEV-Sib has also been found west of the Urals, in the Republic of Komi, alongside TBEV-FE (Mikryukova et al., 2014).

Climate change has led to increased annual and seasonal temperatures, fluctuations in precipitation levels, milder winters, and changes in habitats and the number of host species. This, in turn, has extended the tick activity season, improved their survival during overwintering, and increased tick populations, as well as the spread of pathogens (Medlock et al., 2013; Gilbert, 2021). Recently, there has been a change in tick ranges and TBEV spread to previously non-endemic areas (Heinz et al., 2015; Jahfari et al., 2017; Boelke et al., 2019; Casati Pagani et al., 2019; Makenov et al., 2019; Alfano et al., 2020; Holding et al., 2020; Esser et al., 2022; Gonzalez et al., 2022). The *I. persulcatus* tick population is increasing and moving northward in some European countries (Katargina et al., 2013; Jaenson et al., 2016), as well as in the northeastern European plain in Russia (Tokarevich et al., 2011, Tokarevich et al., 2017; Bugmyrin et al., 2013). The range of *I. ricinus* is also expanding northward (Jaenson et al., 2012; Soleng et al., 2018; Hvidsten et al., 2020; Vikse et al., 2020), with detection of TBEV or antibodies to it in high-altitude areas (Daniel et al., 2004, Daniel et al., 2016; Danielová et al., 2006; Materna et al., 2008; Holzmann et al., 2009; Martello et al., 2014; Kholodilov et al., 2019).

*Ixodes ricinus* and *I. persulcatus* cohabit the East European Plain in Russia, the Baltic countries, and Finland (Bugmyrin et al., 2013; Katargina et al., 2013; Laaksonen et al., 2017). In sympatric zones, there may be a shift in vector species, and some virus subtypes can circulate in non-specific tick species, such as the detection of TBEV-Sib in *I. ricinus* and TBEV-Eu in *I. persulcatus* (Jääskeläinen et al., 2006, Jääskeläinen et al., 2011, Jääskeläinen et al., 2016; Katargina et al., 2013).

Other tick species may also participate in the circulation of TBEV and contribute to maintaining the virus in natural foci. Recent studies have shown that *Dermacentor reticulatus* ticks can act as competent vectors and participate in TBEV circulation in foci alongside *I. ricinus* (Chitimia-Dobler et al., 2019; Ličková et al., 2020). *Dermacentor reticulatus* is widely distributed across European countries from northern Portugal to Western Siberia in Russia (Rubel et al., 2016, Rubel et al., 2020). The geographical distribution of *D. reticulatus* in Europe and European Russia overlaps with the ranges of *I. ricinus* and *I. persulcatus*. In Siberia, *I. persulcatus* coexists with *Ixodes pavlovskyi*, *Dermacentor silvarum*, and *Dermacentor nutalli*, with TBEV detected in all of these tick species (Chausov et al., 2010; Bakhvalova et al., 2016; Tkachev et al., 2017b; Kholodilov et al., 2019). In the Russian Far East, TBEV circulates not only in *I. persulcatus*, but also in *Haemaphysalis concinna*, *Haemaphysalis japonica*, and *Dermacentor silvarum* (Pukhovskaya et al., 2018). *Ixodes ovatus* ticks have also been implicated in TBEV circulation in China (Xing et al., 2017) and Japan (Takeda et al., 1998; Yoshii et al., 2017). In Korea, TBEV has been isolated from *Haemaphysalis longicornis*, *Haemaphysalis flava*, and *Ixodes nipponensis*, with phylogenetic analysis of the E protein gene grouping these strains with the TBEV-Eu (Yun et al., 2012). However, no human cases of TBE have been reported in Korea.

For the viral population to sustain its circulation in nature, it must adapt to replication both in the tick vector and in the vertebrate host. In the tick, the virus must maintain long-term persistence, while in the vertebrate host, rapid virus replication and pronounced viremia are essential. Unlike mammalian cells, in the infected tick cells no cytopathic and ultrastructural changes occur, and persistent infection can be established in these cells (Šenigl et al., 2006; Růžek et al., 2008a; Offerdahl et al., 2012; Weisheit et al., 2015; Belova et al., 2017). The mechanisms of interaction between arboviruses and ticks are only beginning to be explored. It is known that gut epithelial cells are the targets of initial virus infection in ticks (Nuttall and Labuda, 2003). To establish infection in the epithelial cells viruses must overcome several gut barriers. These include physical barriers like peritrophic matrix (PM) (Zhu et al., 1991) and the dityrosine network (DTN) (Yang et al., 2014), gut immunity, and resident microbiota (Yuan et al., 2024). In addition, ticks contain a number of endogenous viruses, which may exhibit interaction with pathogenic tick viruses (Hart and Thangamani, 2021). While such interaction has been observed between viruses in mosquitoes (Goenaga et al., 2015), it has not been demonstrated with viruses in ticks. After escaping from the gut barrier, the arbovirus faces the immune mechanisms of the tick hemolymph, such as antimicrobial peptides (AMPs), phagocytosis, complement-like systems, and coagulation (Yuan et al., 2024). Tick cells are capable of recognizing the presence of pathogens using a variety of surface receptors like Toll receptors and CD36 scavenger receptors (Hart and Thangamani, 2021). In order to infect tissues and cells, viruses must deal with the intrinsic immune response, which include small interfering RNA (siRNA) pathway, Toll pathway, immune deficiency (IMD) pathway, and Janus kinase-signal transduction and activators of transcription (JAK-STAT) pathway (Hart and Thangamani, 2021; Yuan et al., 2024). The role of most of the mentioned pathways in viral infection of ticks is poorly studied. It is thought that the RNAi system appears to be the most critical aspect of tick immunity with regard to viral infection, and that it is aimed at regulating the reproduction of viruses at a certain level, and not at total virus elimination (Hart and Thangamani, 2021). This theory explains the existence of almost lifelong persistent infection of ticks with some arboviruses including TBEV (Nuttall and Labuda, 2003), but the detailed mechanisms of this phenomenon remain to be studied.

Experiments with cell cultures of vector and non-vector tick species have demonstrated that TBEV strain of the European subtype successfully replicates in all cell lines, but in the cells of the *I. ricinus* species typical for TBEV-Eu the titer of the virus was significantly higher than in cell cultures of other tick species (Růžek et al., 2008a). In laboratory experiments using *I. persulcatus*, *I. ricinus*, and *D. reticulatus*, the TBEV-Sib strain successfully replicated and formed a persistent infection in both its specific vector, *I. persulcatus*, and in *I. ricinus* and *D. reticulatus* ticks (Belova et al., 2017).

In special experiment, the mutant TBEV-Sib variants with inserted substitutions characteristic to hemagglutination-defficient variants (including one from patient with encephalitis) isolated in the Yaroslavl region, showed increased replication in the non-specific *I. ricinus* vector. According to these data, adaptation in a non-specific “virus-tick” pair is possible and may lead to the formation of a more virulent virus variant (Khasnatinov et al., 2009). Using chimeric TBEV-Eu (strain Hypr) and TBEV-Sib (strain Vasilchenko) viruses, adaptive characteristics of TBEV were demonstrated in competent tick species, with higher virus titers in the salivary glands of female *I. ricinus* for the characteristic TBEV-Eu strain (Khasnatinov et al., 2016). By using artificially created viral variants, it was possible to trace which mutations or genomic regions are responsible for the mechanism of rapid adaptation.

When assessing morbidity in densely populated regions, it is clear that areas may significantly differ in epidemiological situations. For example, Moscow region borders two TBE-endemic regions (Tver and Yaroslavl), but despite the high population of *I. ricinus* and *I. persulcatus*, TBE cases are almost undetected in Moscow region (Makenov et al., 2019). This is likely due to various factors, one of which could be the characteristics of local tick populations. This assumption is supported by data from Germany, where ticks from natural foci with corresponding TBEV isolates were used. Real-time PCR showed that the likelihood of artificially infecting a tick with TBEV was higher if the ticks were collected from the same location where the TBEV strain had been isolated (Liebig et al., 2021).

In this study, we aim to investigate the properties of the viral population during adaptation to replication in ticks of different species (both specific and non-specific for the virus subtype) and in different populations of the same tick species. Strains of the three main subtypes were used – TBEV-Eu, TBEV-Sib, TBEV-FE – and two geographically distinct populations of primary vectors, *I. ricinus*, *I. persulcatus*, and an additional vector, *D. reticulatus*.

## 2 Materials and methods

### 2.1 Ticks’ collection

Ticks were collected using the flagging method during their peak activity seasons in 2018-2019: *I. ricinus* from the Kaliningrad region (Curonian Spit) (N 55.1591, E 20.8432) and Voronezh region (N 51.6301, E 39.6714), *I. persulcatus* from Republics of Tuva (N 51.3320, E 095.9432) and Karelia (N 62.0635, E 33.9855), and *D. reticulatus* from the Kaluga (N 54.1537, E 35.7495) and Voronezh (N 51.6655, E 39.7562) regions.

Ticks were collected on allopatric territories; species identification was performed using taxonomic keys (Filippova, 1977). The time between collection and experimental procedures did not exceed three weeks. During this period, ticks were stored in tubes with humidity gradient, as described previously (Belova et al., 2012). Briefly, glass tubes were filled with 1/3 of distilled water, and tight cotton swab 2-3 cm in length was inserted in them, so that it nearly all ended up in the water. Filter paper was placed on the top of the cotton swab, and a strip of filter paper was inserted in each tube to allow ticks migrate in tubes according to the preferred humidity. All tubes were tightly closed with cotton-gauze cap.

### 2.2 Viruses and cells

In the present study, three strains of the main TBEV subtypes were used from the laboratory of arboviruses’ collection of the Chumakov FSC R&D IBP RAS (Institute of Poliomyelitis) (**Table 1**). Viruses were derived from the supernatants of infected cell cultures. Additionally, the attenuated poliovirus type I Sabin strain served as an internal control during RNA isolation.

**Table 1:** Characteristics of TBEV strains used in the study.

TBEV Strain	Subtype	Origin and Year	Source	Passage history	GenBank ID	Titer* (log PFU/mL)	RNA Copies (log GCN/mL)
LK-138	TBEV-Eu	Lithuania, 1972	adult *I. ricinus*	М_2_Р_4_	GU125720	6.8 ± 0.3	9.2 ± 0.2
Karl08-T3522	TBEV-Sib	Republic of Karelia, Russia, 2008	adult *I. persulcatus*	М_3_V_1_	KU052689	6.4 ± 0.3	9.5 ± 0.2
DV-936k	TBEV-FE	Primorsky Krai, Russia, 1975	adult *H. concinna*	М_2_P_3_	GU125722	6.7 ± 0.3	9.7 ± 0.2

М, passages in white mice brain; P, passages in PEK cell line; V, passage in Vero cell line; PFU, plaque-forming unit; GCN, genome copy number; *, in the culture supernatant of infected PEK cells.

In this paper, we assumed that specific tick population of the used TBEV-Eu strain is *I. ricinus* from the Curonian Spit, specific tick population of the used TBEV-Sib strain is *I. persulcatus* from the Republic of Karelia, and of the TBEV-FE strain – *I. persulcatus* from the Republic of Tuva. Other *Ixodes* ticks populations and both populations of *D. reticulatus* were supposed to be non-specific ticks for the TBEV strains used in this study.

For simplicity, abbreviations were adopted: LK-138 as LK-Eu, Karl08-T3522 as Karl-Sib, and DV-936k as DV-FE.

Porcine embryo kidney (PEK) cell line was maintained at 37°C in medium 199 with Hanks’ balanced salt solution and Earle’s balanced salt solution (2:1, v:v, FSASI “Chumakov FSC R&D IBP RAS”, Moscow, Russia) supplemented with 5% fetal bovine serum (FBS, Gibco, USA). Vero cell line was maintained at 37°C in DMEM medium supplemented with L-glutamine (FSASI “Chumakov FSC R&D IBP RAS”, Moscow, Russia) and 10% FBS (Gibco, USA).

### 2.3 Experimental infection of ticks

Ticks were percoxally infected according to the method described earlier (Belova et al., 2012). Briefly, ticks were immobilized by fixing their ventral surface up to the vacuum holder and under the binocular virus suspension was injected in the joint of the tick coxa and trochanter of the 4th pair of legs. Ticks were infected with TBEV at equivalent multiplicity of infection based on the size of the ticks. In case of mono-infection, 1 μL of virus-containing fluid was inoculated in *D. reticulatus* ticks (infection dose of the strain LK-Eu was 3.8 ± 0.3 log plaque-forming unit (PFU), 6.2 ± 0.2 log genome copy number (GCN); Karl-Sib – 3.4 ± 0.3 logPFU, 6.5 ± 0.2 logGCN; DV-FE – 3.7 ± 0.3 logPFU, 6.7 ± 0.2 logGCN), and 0.5 μL – in *Ixodes* ticks (infection dose of the strain LK-Eu was 3.5 ± 0.3 logPFU, 5.9 ± 0.2 logGCN; Karl-Sib – 3.1 logPFU ± 0.3, 6.2 ± 0.2 logGCN; DV-FE – 3.4 logPFU ± 0.3, 6.4 ± 0.2 logGCN). In experiments with mixed infection, ticks were injected with a mixture of strains LK-Eu and Karl-Sib; infection dose for *D. reticulatus* was 5.9 ± 0.2 logGCN and 6.2 ± 0.2 logGCN, and for *Ixodes* ticks – 5.6 ± 0.2 logGCN and 5.9 ± 0.2 logGCN, respectively. After infection, ticks were placed in tubes with gradient humidity and at certain time points post infection (p.i.) 3-5 individuals were selected and individually frozen; if there were dead or half-dead individuals at a certain point, they were also frozen. The end point of the experiment depended on the survival of ticks. In each group, the same number of ticks was initially selected. Ticks were stored at -70°C and homogenized before analysis.

### 2.4 Sample preparation

Ticks were individually suspended in 500 μL of Medium 199 (FSASI “Chumakov FSC R&D IBP RAS,” Russia). Suspensions were prepared as 0.17% for *Ixodes* (average tick weight ~0.85 mg) and 0.7% for *D. reticulatus* (average weight ~3.5 mg). Concentration differences were taken into account during data analysis, and results were normalized to a 0.17% suspension. Homogenization was performed with a TissueLyser II (QIAGEN, Germany) at 25 Hz for 10 minutes at room temperature, using a single stainless steel bead (d = 7 mm) per tube. Following bead removal, suspensions were centrifuged at 1500 rpm for 5 minutes. Aliquots of 125 μL were used for RNA extraction, 50 μL for titration in PEK cell culture, and the remainder stored at -70°C.

### 2.5 Plaque assay in PEK cells

Virus titration was conducted on 6-well plates (Corning, NY, USA) with PEK cell monolayers under an agar overlay, as described previously (Belova et al., 2012). Titer values were expressed as logPFU/mL of the 0.17% tick suspension.

### 2.6 RNA isolation, reverse transcription

RNA was extracted from 125 μL of tick suspension using TRI reagent (Sigma-Aldrich, USA), following the manufacturers’ protocol. A fixed amount (5.5 log RNA copies per sample) of the attenuated poliovirus type I Sabin strain was added as an internal control. Reverse transcription employed M-MLV reverse transcriptase (Promega, USA). For mono-infections, a single reaction tube contained primers targeting TBEV and poliovirus (**Supplementary Table S1**). For mixed infections, three separate reactions were conducted for LK-Eu, Karl-Sib, and poliovirus strains.

### 2.7 Quantitative Real-Time PCR (qPCR)

#### 2.7.1 Mono-infection

qPCR was performed as described earlier (Tuchynskaya et al., 2021). Amplification was carried out on C1000 Touch Thermal Cycler with CFX96 Optical Reaction Module (BioRad, USA) using RT-PCR kit R-412 (Syntol, Russia) according to protocol: 95°C – 5 min, 42 cycles: 60°C –45 s, 95°C – 15 s. Fluorescence was recorded at 60°C in FAM and ROX channels. The standard curve was established using serial dilutions of fragment of TBEV RNA, obtained through *in vitro* transcription of PCR-product (**Supplementary Table S1**) followed by purification in the gradient of sucrose (Litov et al., 2023). Quantitative PCR on the 3’-non-coding region (3’-NTR) of the TBEV was performed using primers R-TBE, F-TBE, and probe TBE-probe (**Supplementary Table S1**). For internal control, quantitative PCR on the 3Dpol of the poliovirus was conducted using primers PVR1, PVL1, and probe PVP (**Supplementary Table S1**). Viral RNA quantity in the samples was expressed as a decimal logarithm of the GCN per mL of the 0.17% tick suspension (logGCN/mL).

#### 2.7.2 Mixed infection

The process of separate quantitative detection of the strains LK-Eu and Karl-Sib in mixture was similar to that of the mono-infection with some modifications. Based on the RT-PCR system described earlier (Karan et al., 2007), primers and probes for the strains LK-Eu and Karl-Sib were developed (**Supplementary Table S1**). The PCR conditions were 95°C for 5 min, followed by 50 cycles at 95°C for 15 s, 60°C for 30 s, and 72°C for 20 s. The fluorescent signal was recorded at 60°C in the FAM and ROX channels in separate tubes. Standard curves of the strains LK-Eu and Karl-Sib were generated as described above using strain-specific primer pairs (**Supplementary Table S1**). The limit of detection for this method was determined by serial dilutions to be 60 RNA copies of the TBEV strains per PCR reaction tube, so the limit of quantification (threshold) was set as 100 RNA copies per PCR reaction (2.5×10^4^ copies per mL of the sample). To test the specificity of the assay, we tested different concentrations of the strains LK-Eu and Karl-Sib, separately and in mix (**Supplementary Table S2**). According to the obtained data, the selected system quite accurately determined the number of copies of the target strain and with the absence of non-specific interactions.

### 2.8 Statistical analysis

Data were analyzed using OriginPro 8 SR4 (v8.0951, Northampton, MA, USA). Comparisons were made using the Mann-Whitney U-test for two groups. Differences with p < 0.05 were considered statistically significant. Bonferroni corrections were applied for multiple comparisons. Heatmaps and graphs were generated in GraphPad Prism 9 (GraphPad Software Inc., San Diego, CA, USA).

## 3 Results

### 3.1 Characterization of the virus during persistent infection in ticks

In order to test the hypothesis that the properties of the “tick-virus” pair are unique to a specific tick species, ticks of each species were collected from two geographically distant regions. All tick populations were infected with the TBEV (LK-Eu, Karl-Sib, DV-FE) to establish persistent infection. At predetermined time points following infection, the level of virus replication was evaluated by the number of TBEV RNA copies using real-time PCR and by the number of infectious virus particles for mammalian cells using plaque assay in PEK cell culture. The infectivity of the virus was assessed by the ratio of RNA copies to plaque-forming units (logGCN - logPFU): a higher ratio indicates lower infectivity.

#### 3.1.1 Determination of infectious virus titers

**Figure 1:**
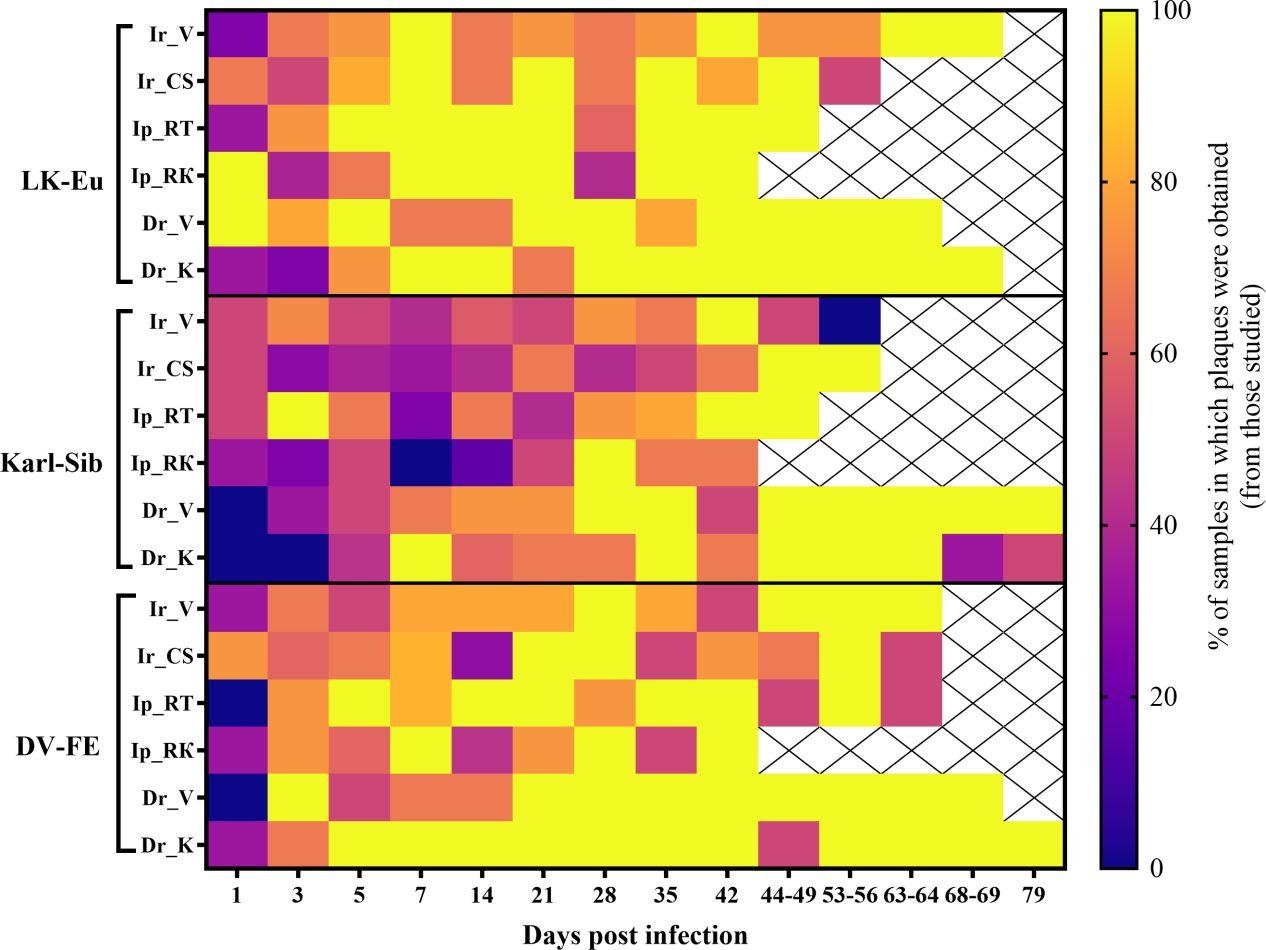
Heatmap of TBEV infectivity. Presence of infectious virus > 1.4 log PFU/mL of 0.17% suspension of tick infected with TBEV (strains LK-Eu, Karl-Sib, DV-FE), where 100% is indicated by yellow, and 0% by blue. Groups: Ir_V, *Ixodes ricinus*, Voronezh; Ir_CS, *I. ricinus*, Curonian Spit; Ip_RT, *I. persulcatus*, Republic of Tuva; Ip_RK, *I. persulcatus*, Republic of Karelia; Dr_V, *Dermacentor reticulatus*, Voronezh; Dr_K, *D. reticulatus*, Kaluga.

During the course of the experiments, difficulties were encountered in the titration of certain samples. Despite the presence of relatively high RNA copy numbers, the titer of the infectious virus was below the minimum sensitivity of the method employed (<1.4 log PFU/mL of 0.17% tick suspension). This was particularly evident for the strain Karl-Sib. In **Figure 1** a heatmap illustrates the percentage of samples that were positive (yellow color, 100%) and negative (dark color) in the plaque assay.

Plaque titration was performed by the same researcher utilizing standard cell cultures and materials, thereby minimizing the potential for experimental or methodological errors.

For further calculations of infectivity, the virus titer in negative in plaque assay samples was assumed to be 1 logPFU.

#### 3.1.2 Plaques phenotype in TBEV-infected PEK cell line

The plaque phenotype in cell culture under standard conditions has been shown to serve as a distinct genetic trait of the virus. The emergence of plaques with altered phenotypes within the population signifies the occurrence of variants that possess novel properties.

During the adaptation of the viral population to ticks, changes in the plaque phenotype in mammalian PEK cell culture were observed. On the seventh day of observation, the plaques were classified into five groups: 1 – small pinpoint plaques, 2 – plaques 1-1.5 mm in size, 3 – plaques 2-4 mm in size, 4 – plaques larger than 4 mm, and 5 – plaques of various sizes with indistinct, irregular star-shaped contour (**Supplementary Figure S1**). **Table 2** represents the plaque phenotype of TBEV variants: bold numbers indicate the predominant plaque phenotype, and italic numbers in parentheses indicate the minor phenotype.

**Table 2:** Representation of plaque phenotype variants in PEK cell culture (day 7) during plaque assay under an agar overlay for TBEV variants obtained during persistence in ticks.

Strain	Days p.i.	*I. ricinus*		*I. persulcatus*		*D. reticulatus*	
		Voronezh	Curonian Spit	Tuva	Karelia	Voronezh	Kaluga
LK-Eu **phenotype 3 (50%), phenotype 4 (50%)**	3-7	**2,4** *(1,3)*	**2,3** (-)	**1,2,3** (-)	**2,3** *(1,4)*	**2,3** (-)	**2,3** *(1)*
	14-21	**2,3** (-)	**2,3** *(4)*	**1,2,3** (-)	**1,2** (-)	**1,3** *(2,4)*	**1,2,3** (-)
	28-42	**2,3** *(1,4)*	**2,3** *(4)*	**2,3** *(1)*	**1,2** (-)	**1,2,3,4** (-)	**1,2** *(3,4,5)*
	46-53	**2** *(1)*	**3** (-)	**3** *(2,4)*	nd	**1,2** (-)	**1,2** *(3)*
	64-70	**1** *(2,3)*	nd	nd	nd	**1** (-)	nd
Karl-Sib **phenotype 2 (75%),** *phenotype 3 (25%)*	3-7	**1,2** (-)	**1** (-)	**1,2,3** (-)	**5** (-)	**1,4** (-)	**4** (-)
	14-21	**1** (-)	**1,3** (1)	**1,2** *(3)*	**1,2** (-)	**2,3** *(1)*	**2,3,4** (-)
	28-42	**1,2** *(5)*	**1,2,3** (-)	**1,3** (-)	**2,3,4,5** *(1)*	**1,2,5** *(3)*	**1,2,4** *(3)*
	45-56	**3** (-)	**1,2** (-)	nd	nd	**2,3,5** *(1)*	**3** *(2/5)*
	63-79	nd	nd	nd	nd	**3** (-)	**2,3** (-)
DV-FE *phenotype 3 (20%)*, **phenotype 4 (80%)**	3-7	**1,2,3,4** (-)	**3,4** (-)	**2,3** (-)	**3,4** (-)	**2,3,4** (-)	**2,3,4** (-)
	14-21	**1,2,3,4** (-)	**3** (-)	**2** *(1)*	**2,3** *(4)*	**2,4** *(3)*	**4** (-)
	28-42	**2,3** *(1)*	**2,4** *(1,3)*	**2,4** *(1,3)*	**2,3,4** *(1)*	**2,3,4** (-)	**3,4** (-)
	44-45	**2** (-)	**2,4** (-)	**2,3** (-)	nd	nd	**2,4** *(1,3)*
	63-69	**2** (-)	nd	nd	nd	**1,2** (-)	**2,3,4** (-)

Bold, most commonly observed phenotype variant (100% in the sample or the highest percentage in cases of heterogeneity); *Italics*, phenotype variant observed in cases of heterogeneity (lower percentage); “–”, no minor variant; nd, no data.

For the original LK-Eu strain, a heterogeneous population of plaques with phenotypes 3 (50%) and 4 (50%) was observed in PEK cells. For variants that persisted in *D. reticulatus* ticks, the highest number of different plaque phenotypes was recorded, irrespective of the geographical origin of the tick populations. With the exception of *I. ricinus* population from the Curonian Spit (specific vector for the TBEV-Eu), phenotype 1 (small plaques) was observed in all other populations. Thus, phenotype 4, characteristic to 50% of plaques of the original strain LK-Eu, practically disappears during virus adaptation to persistent infection in ticks (**Table 2**).

For the original Karl-Sib strain, the plaque phenotypes 2 and 3 were predominant in PEK cell culture in a ratio of 75% and 25%, respectively. As previously mentioned, many samples, especially from the early time points, did not form plaques in PEK cell culture (**Figure 1**). For variants positive in plaque assay, the plaques were faint on day 5, becoming more noticeable by day 7. Following a persistence period of over 42 days in *D. reticulatus*, the number of samples positive in plaque assay increased. For the *D. reticulatus* population from Voronezh region, variants with phenotype 1 were observed. Additionally, larger phenotype 4 plaques were noted in *D. reticulatus* at different time points, a feature not observed in the two *I. ricinus* populations or the *I. persulcatus* population from the Republic of Tuva. The greatest diversity of the Karl-Sib plaque phenotypes, as with LK-Eu, was observed in *D. reticulatus* ticks.

Phenotypes 3 (20%) and 4 (80%) were characteristic of the DV-FE strain in PEK cell culture. In the tick population from the Voronezh region, variants with small plaque sizes accumulated: in *I. ricinus*, phenotype 2 appeared, and in *D. reticulatus*, phenotypes 1 (50%) and 2 (50%) were observed. In the other tick populations, the viral phenotype was represented by a combination of 3,4 or 2,3.

Consequently, during persistence in ticks of all virus species, especially in the early stages of infection, an increase in population heterogeneity by plaque size was observed in PEK cell culture, manifested by the appearance of small plaques (<1 mm and 1-1.5 mm).

#### 3.1.3 Dynamics of TBEV strains reproduction in different local tick populations

**Figure 2:**
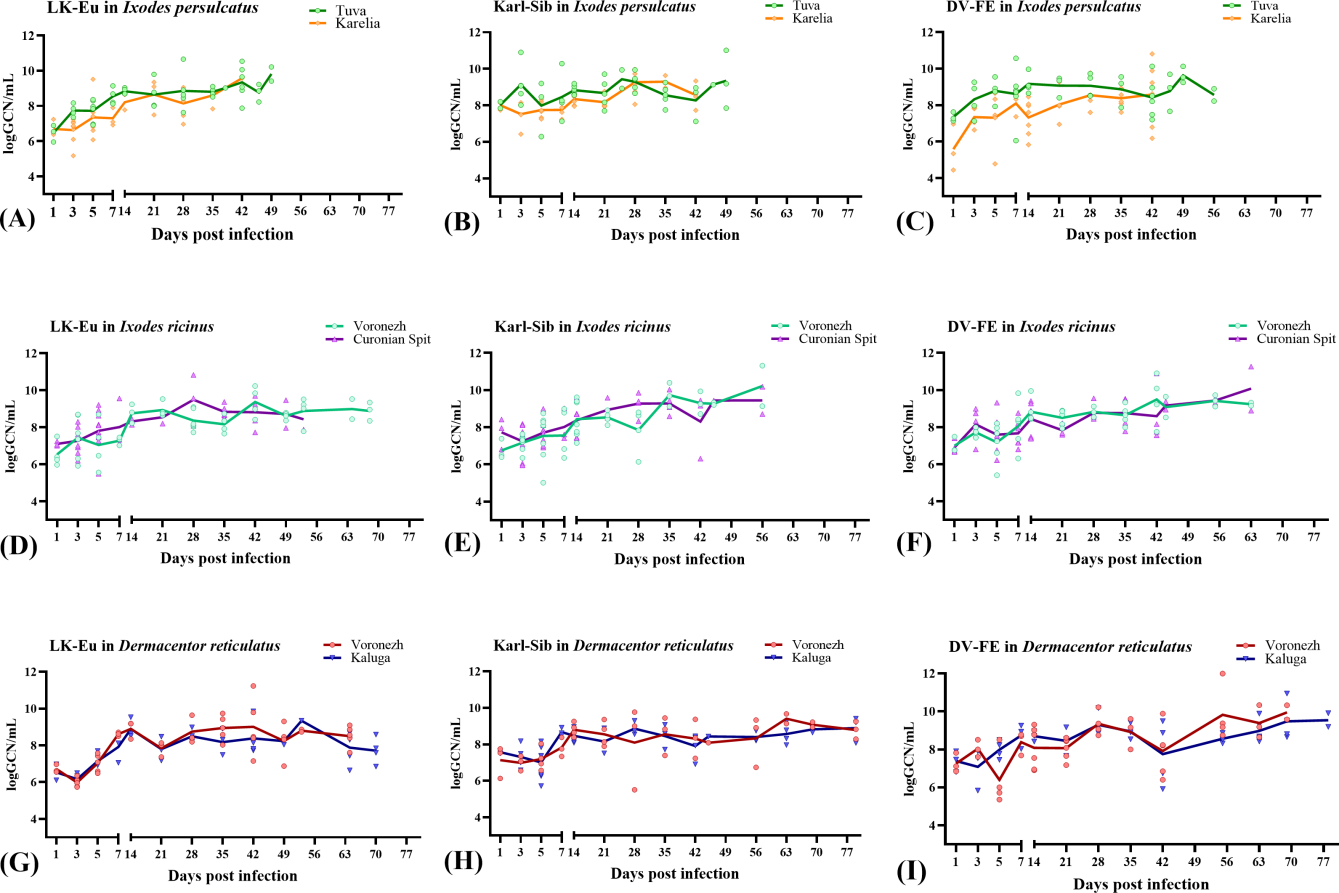
Reproduction level of the TBEV strains LK-Eu **(A, D, G)**, Karl-Sib **(B, E, H)**, and DV-FE **(C, F, I)** in two geographical populations of ticks. *Ixodes persulcatus* **(A–C)**: Tuva - green, Karelia – orange; *Ixodes ricinus* **(D–F)**: Voronezh - turquoise, Curonian Spit – purple; *Dermacentor reticulatus* **(G–I)**: Voronezh - blue, Kaluga - red.

**Figure 3:**
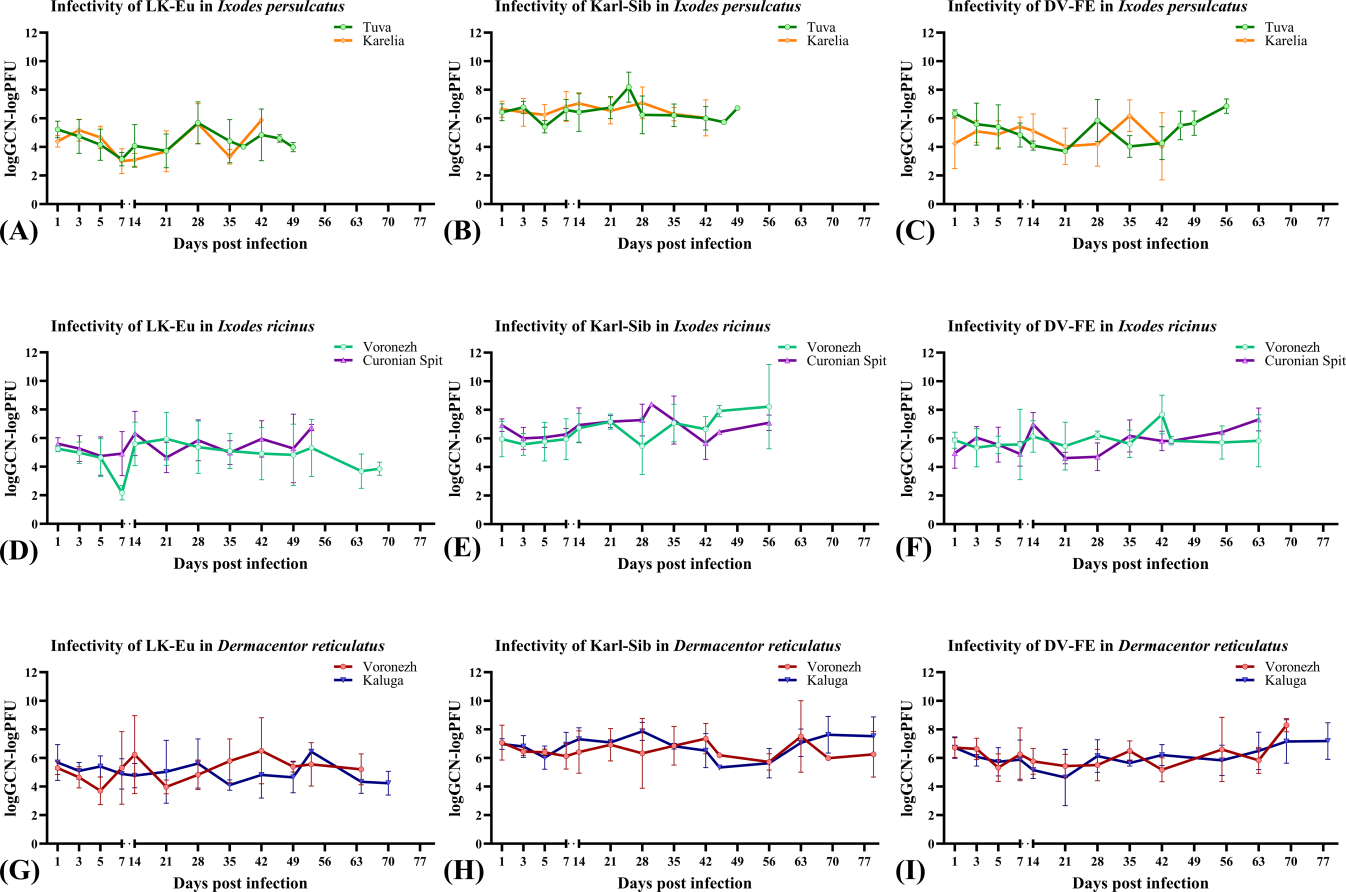
Infectivity of the TBEV strains LK-Eu **(A, D, G)**, Karl-Sib **(B, E, H)**, and DV-FE **(C, F, I)** in two geographical populations of ticks. *Ixodes persulcatus* **(A–C)**: Tuva - green, Karelia – orange; *Ixodes ricinus* **(D–F)**: Voronezh - turquoise, Curonian Spit – purple; *Dermacentor reticulatus* **(G–I)**: Voronezh - blue, Kaluga – red.

**Figures 2**, **3** show the dynamics of virus reproduction based on the data on viral genome copy number and the change in its infectivity throughout the experiment. The amount of infectious virus and the ratio of GCP to PFU were determined only for those samples where the infectious virus was detected. For each tick population, the end point is different due to the different survival of ticks. When comparing groups using the Mann-Whitney test, data up to the day common to the compared groups was used.

The reproduction level of the strains LK-Eu, Karl-Sib, and DV-FE in *I. persulcatus* ticks from Tuva was significantly higher compared to that in ticks from Karelia (Mann-Whitney test, p=0.001; p=0.01; p=0.0008, respectively) (**Table 3**; **Figures 2A–C**). When comparing the infectivity of the TBEV for mammalian cell line between the *I. persulcatus* populations, no significant differences were found for any of the strains (**Table 3**; **Figures 3A–C**). Thus, despite the higher reproduction level of all TBEV strains in the *I. persulcatus* population from Tuva, compared to the population from Karelia, the infectivity of the virus in the two tick populations was similar.

**Table 3:** Comparison of characteristics of TBEV strains in different populations of ticks of the same species (Mann-Whitney test).

Tick species	group 1	group 2	days p.i.	p (reproduction rate)	p1 (infectivity)
*I. ricinus*	(Voronezh) LK-Eu	(Curonian Spit) LK-Eu	1-53	0.79072	0.16301
	(Voronezh) Karl-Sib	(Curonian Spit) Karl-Sib	1-56	0.33528	0.24306
	(Voronezh) DV-FE	(Curonian Spit) DV-FE	1-63	0.53602	0.86628
*I. persulcatus*	(Tuva) LK-Eu	(Karelia) LK-Eu	1-42	0.00173	0.72102
	(Tuva) Karl-Sib	(Karelia) Karl-Sib	1-42	0.01475	0.67576
	(Tuva) DV-FE	(Karelia) DV-FE	1-42	8.78E-04	0.81652
*D. reticulatus*	(Kaluga) LK-Eu	(Voronezh) LK-Eu	1-64	0.28358	0.64948
	(Kaluga) Karl-Sib	(Voronezh) Karl-Sib	1-63	0.77066	0.50559
	(Kaluga) DV-FE	(Voronezh) DV-FE	1-63	0.65869	0.70966

Significant differences are highlighted in color, taking into account the significance level of 0.05.

For *I ricinus* and *D. reticulatus* ticks, the reproduction level and infectivity of all TBEV strains were similar for the two geographic populations of different tick species (**Table 3**; **Figures 2D–I**; **Figures 3D–I**).

We were able to reveal geographic differences in *I. persulcatus* populations regarding the dynamics of RNA copy accumulation of the studied TBEV strains.

#### 3.1.4 Reproduction of TBEV strains in different tick species

**Figure 4:**
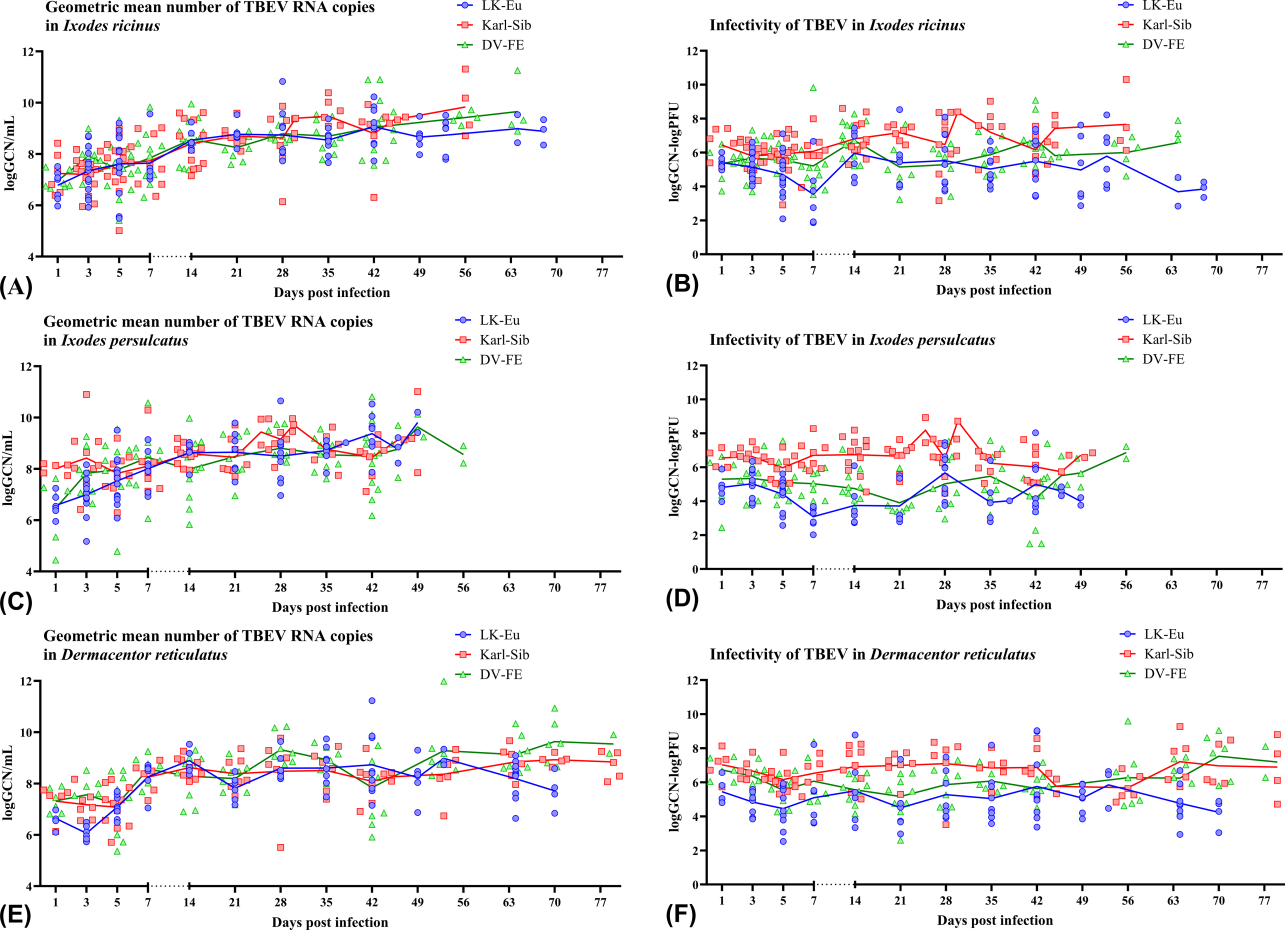
Replication level and infectivity of TBEV strains LK-Eu (blue line), Karl-Sib (red line), and DV-FE (green line) during persistent infection of *Ixodes ricinus* **(A, B)**, *Ixodes persulcatus* **(C, D)**, and *Dermacentor reticulatus* **(E, F)**. **(A, C, E)** geometric mean number of RNA copies (logGCN/mL); **(B, D, F)** infectivity, expressed as the decimal logarithm of the ratio of RNA copies to the titer of infectious virus (logGCN - logPFU).

To assess the ability of different tick species to support the reproduction of TBEV strains, data obtained for two populations was combined to increase the sample size and average the values. During the observation period, all TBEV strains demonstrated similar reproduction levels in *I. ricinus* (**Figure 4A**), but differed significantly in infectivity for mammalian cell line (**Figure 4B**; **Table 4**). Karl-Sib was the least infective. The infectivity of the LK-Eu and DV-FE strains also differed significantly (Mann-Whitney test, p1 = 0.001, **Table 4**). The highest infectivity was observed for LK-Eu at 7 days p.i., although the GCN for the three strains did not differ. The highest number of RNA copies was observed for DV-FE at 35 days p.i., which did not affect the infectivity values. Thus, at the same level of reproduction in *I. ricinus* ticks, the LK-Eu strain had greater infectivity for mammalian cell culture compared to the Karl-Sib and DV-FE strains.

**Table 4:** Comparison of the ability of different tick species to support the reproduction of TBEV strains (Mann-Whitney test).

Tick species	group 1	group 2	days p. i.	p (reproduction rate)	p1 (infectivity)
*I. ricinus* (Voronezh + Curonian Spit)	LK-Eu	Karl-Sib	1-53/56	0.6184	2.53E-10
	LK-Eu	DV-FE	1-53/55	0.5156	0.00116
	Karl-Sib	DV-FE	1-56/55	0.87081	1.40E-04
*I. persulcatus* (Karelia + Tuva)	LK-Eu	Karl-Sib	1-42	0.01941	1.99E-20
	LK-Eu	DV-FE	1-42	0.33681	0.04061
	Karl-Sib	DV-FE	1-42	0.20094	4.66E-15
*D. reticulatus* (Voronezh + Kaluga)	LK-Eu	Karl-Sib	1-64/63	0.51171	5.33E-15
	LK-Eu	DV-FE	1-64/63	0.01664	1.13E-06
	Karl-Sib	DV-FE	1-63	0.07428	2.64E-05

Significant differences are highlighted in color, taking into account the significance level of 0.015 (Bonferroni correction).

Similar results were obtained in *I. persulcatus* ticks. With similar reproduction levels, the strain Karl-Sib had the lowest infectivity, and significantly differed from the strains LK-Eu and DV-FE (**Table 4**; **Figures 4C, D**). However, the infectivity of LK-Eu and DV-FE did not differ significantly (**Table 4**). During reproduction of TBEV in *I. persulcatus*, the accumulation of RNA copies occurred from 1 to 7 days p.i., then increased at 28 days p.i., and the next increase occurred at 49 days p.i. (for Karl-Sib and DV-FE, for LK-Eu there are no data on 49 days p.i.). Similar picture was observed in *D. reticulatus* ticks. The strains did not differ in reproduction level, the least infectious was Karl-Sib, and the most infectious was LK-Eu (**Figures 4E, F**).

The presented data showed that all tick species maintained the reproduction of all TBEV strains at approximately the same level. At the same time, the infectivity of the viruses varied significantly. The lowest infectivity of TBEV in the three tick species was characteristic for the strain Karl-Sib and the highest − for LK-Eu, but only in *I. ricinus* and *D. reticulatus.*

When comparing TBEV strains reproduction in different tick species, the combined data for the two tick populations of each species was used.

All tick species effectively supported the reproduction of the strain LK-Eu, and the dynamics of the accumulation of RNA copies of the virus did not differ significantly between tick species (**Supplementary Figure S2A**; **Table 5**). The highest values of the LK-Eu GCN were observed in *I. ricinus*, while a higher proportion of infectious virus of the strain was in *I. persulcatus*, which are not typical for this TBEV subtype (**Supplementary Figure S2B**; **Table 5**; Mann-Whitney test p1 = 0.001).

**Table 5:** Comparison of characteristics of TBEV strains, which persisted in ticks of different species (Mann-Whitney test).

TBEV strain	group 1	group 2	days p.i.	p (reproduction rate)	p1 (infectivity)
LK-Eu	*I. ricinus* (Voronezh+Curonian Spit)	*I. persulcatus* (Karelia+Tuva)	1-42	0.82288	0.00187
	*I. persulcatus* (Karelia+Tuva)	*D. reticulatus* (Voronezh+Kaluga)	1-42	0.346	0.02822
	*I. ricinus* (Voronezh+Curonian Spit)	*D. reticulatus* (Voronezh+Kaluga)	1-64	0.11507	0.51377
Karl-Sib	*I. ricinus* (Voronezh+Curonian Spit)	*I. persulcatus* (Karelia+Tuva)	1-5	0.00437	0.04227
			7-42	0.55483	0.38138
	*I. persulcatus* (Karelia+Tuva)	*D. reticulatus* (Voronezh+Kaluga)	1-5	2.04E-04	0.92635
			7-42	0.84509	0.01111
	*I. ricinus* (Voronezh+Curonian Spit)	*D. reticulatus* (Voronezh+Kaluga)	1-5	0.22889	0.02732
			7-42	0.29284	0.15754
DV-FE	*I. ricinus* (Voronezh+Curonian Spit)	*I. persulcatus* (Karelia+Tuva)	1-56	0.66106	6.05E-05
	*I. persulcatus* (Karelia+Tuva)	*D. reticulatus* (Voronezh+Kaluga)	1-56	0.70388	1.84E-05
	*I. ricinus* (Voronezh+Curonian Spit)	*D. reticulatus* (Voronezh+Kaluga)	1-56	0.92123	0.60235

Significant differences are highlighted in color, taking into account the significance level of 0.015 (Bonferroni correction).

The results obtained for the strain Karl-Sib were different. During reproduction in *I. persulcatus* (specific vectors of this TBEV subtype), the number of RNA copies in the period 1-5 days p.i. significantly differed from the values for *I. ricinus* and *D. reticulatus* ticks (**Table 5**; Mann-Whitney test, p<0.015). At the following dates, the reproduction level was approximately the same for all tick species. Infectivity for mammalian cell line of the Karl-Sib did not differ in all tick species.

The reproduction level of DV-FE did not differ in all tick species (**Supplementary Figure S2E**). The highest infectivity of this strain was observed in *I. persulcatus* ticks (**Table 5**, significant difference from *D. reticulatus*, Mann-Whitney test, p1 = 0.00001; from *I. ricinus*, Mann-Whitney test, p1 = 0.00001), while in *I. ricinus* and *D. reticulatus* ticks this indicator was approximately at the same level (**Supplementary Figure S2F**).

Thus, the obtained results demonstrated that the reproduction level of TBEV strains of different subtypes is approximately equal in all tick species, with the exception of the initial stages of reproduction of Karl-Sib in its specific tick – *I. persulcatus*. The process of selection of a more infectious TBEV variant during reproduction in ticks occurs differently and depends on the virus strain and tick species.

### 3.2 Mixed infection of ticks with the strains LK-Eu and Karl-Sib

Two populations *I. ricinus*, *I. persulcatus*, and *D. reticulatus* were infected with a mixture of the strains LK-Eu and Karl-Sib. The number of TBEV RNA copies was estimated at certain time points using a developed real-time PCR system. The nature of the interaction of the strains in ticks was assessed based on the ratio of the of RNA copies number of the strain LK-Eu to the Karl-Sib. A comparison of the reproduction level of strains during mono- and mixed infection was also conducted.

#### 3.2.1 Level of the TBEV strains reproduction during mixed infection

**Figure 5:**
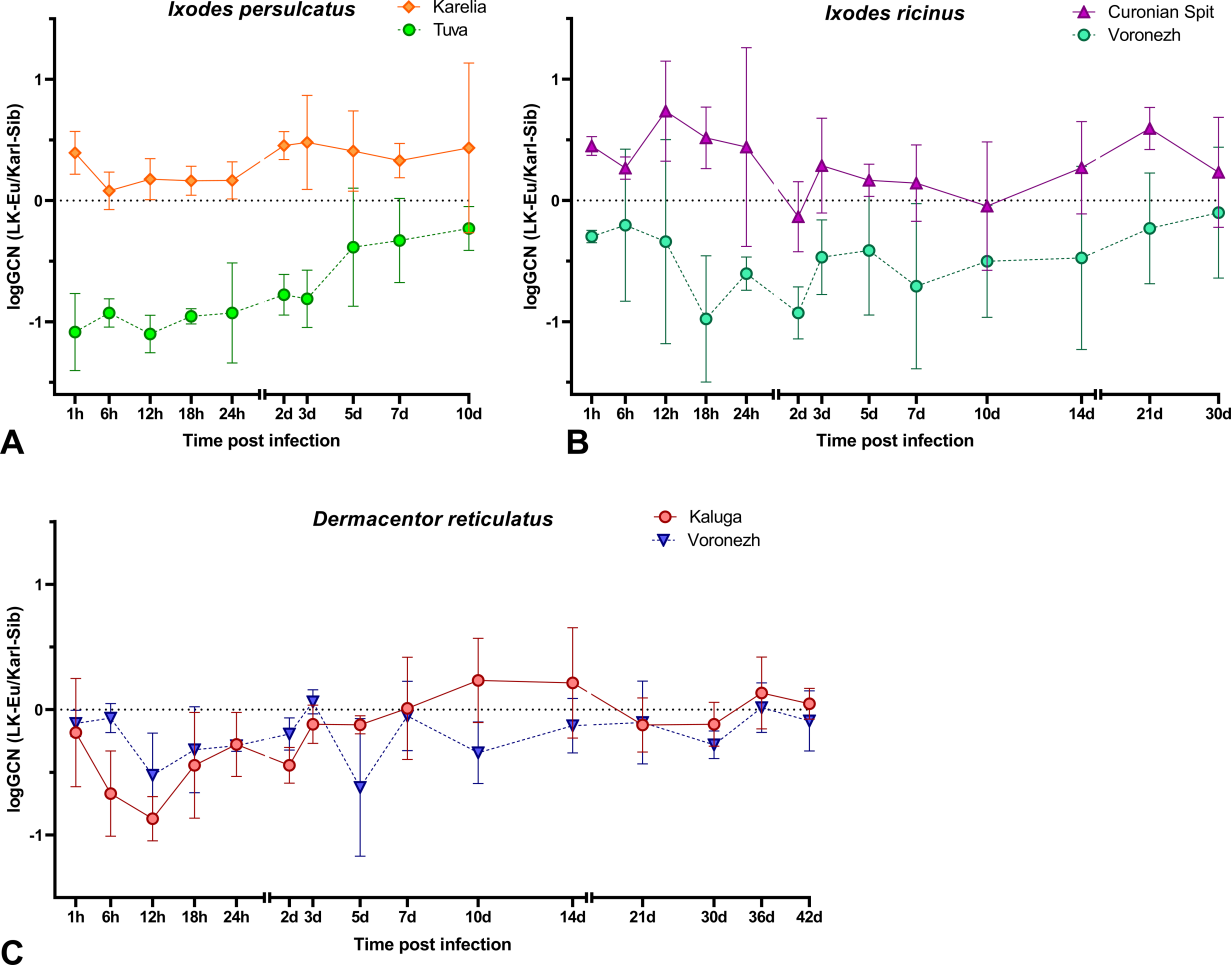
The ratio of the RNA copies number of the LK-Eu and Karl-Sib strains (logGCN(LK-Eu/Karl-Sib)) in a mixed infection of **(A)** *Ixodes persulcatus* from the Republics of Tuva and Karelia, **(B)** *Ixodes ricinus* from the Curonian Spit and Voronezh Region, **(C)** *Dermacentor reticulatus* from Kaluga and Voronezh Regions.

In *I. persulcatus* from Karelia and *I. ricinus* from Curonian Spit, the ratio of the strains in a tick was close to the same or with a slight dominance of LK-Eu (**Figures 5A, B**); the reproduction level of both strains throughout the observation period was similar in *I. ricinus* (**Supplementary Figures S3C, D**; **Table 6**) and *I. persulcatus* (**Supplementary Figures S3A, B**; **Table 6**). In *I. persulcatus* from Tuva and *I. ricinus* from Voronezh region, the Karl-Sib strain dominated throughout the observation period (**Figures 5A, B**; **Table 6**). The dynamics of LK-Eu and Karl-Sib strains reproduction in ticks differed not only between the strains, but also between tick populations of the same species. The reproduction level of the LK-Eu and Karl-Sib strains in different *Ixodes* tick populations differed significantly (**Supplementary Figures S3A-C**; **Table 6**), with the exception of *I. ricinus*, in which the dynamics of the Karl-Sib RNA copies accumulation did not differ between populations from the Curonian Spit and from the Voronezh region (**Supplementary Figure S3D**).

**Table 6:** Comparison of the LK-Eu and Karl-Sib strains reproduction during mixed infection of tick populations of different species (Mann-Whitney test).

Tick species/place of collection	group 1		group 2	Mann-Whitney test, р
Ratio of the RNA copies number of the LK-Eu and Karl-Sib, logGCN(LK-Eu/Karl-Sib)				
*I. persulcatus*	(Tuva) LK/Karl	<	(Karelia) LK/Karl	<0,0001
*I. ricinus*	(Voronezh) LK/Karl	<	(Curonian Spit) LK/Karl	<0,0001
*D. reticulatus*	(Voronezh) LK/Karl		(Kaluga) LK/Karl	0,4845
Level of reproduction of the LK-Eu and Karl-Sib in ticks, logGCN/mL				
*I. persulcatus*	(Tuva) LK-Eu	<	(Karelia) LK-Eu	0,0039
	(Tuva) Karl-Sib	>	(Karelia) Karl-Sib	0,00459
	(Karelia) Karl-Sib		(Karelia) LK-Eu	0,0325
	(Tuva) Karl-Sib	>	(Tuva) LK-Eu	0,00036
*I. ricinus*	(Curonian Spit) Karl-Sib		(Curonian Spit) LK-Eu	0,1403
	(Voronezh) Karl-Sib	>	(Voronezh) LK-Eu	0,0072
	(Voronezh) LK-Eu	<	(Curonian Spit) LK-Eu	<0,0001
	(Voronezh) Karl-Sib		(Curonian Spit) Karl-Sib	0,9404
*D. reticulatus*	(Kaluga) Karl-Sib		(Kaluga) LK-Eu	0,5354
	(Voronezh) Karl-Sib		(Voronezh) LK-Eu	0,1938
	(Voronezh) LK-Eu		(Kaluga) LK-Eu	0,6219
	(Voronezh) Karl-Sib		(Kaluga) Karl-Sib	0,9764
Level of reproduction of the LK-Eu and Karl-Sib in ticks from Voronezh region, logGCN/mL				
Voronezh region	*I. ricinus* Karl-Sib	>	*D. reticulatus* Karl-Sib	0,0002
	*I. ricinus* LK-Eu		*D. reticulatus* LK-Eu	0,0381

Significant differences are highlighted in color, taking into account the significance level of 0.0125 (Bonferroni correction).

In *D. reticulatus* populations, the reproduction dynamics of the LK-Eu and Karl-Sib strains did not differ statistically (**Supplementary Figures S3E, F**; **Table 6**), and the ratio of strains in ticks starting from 24 h after infection was almost the same (**Figure 5C**; **Table 6**). Comparison of the dynamics of the TBEV RNA copies accumulation in ticks from the Voronezh region showed that the reproduction level of the strain Karl-Sib in *I. ricinus* was significantly higher than in *D. reticulatus*, and reproduction of the strain LK-Eu did not differ in these two tick species (**Supplementary Figures S3D, F**; **Table 6**).

Interestingly, in almost all experimental groups, at the end point of observations, the ratio of the number of copies of strains in ticks was close to 1.

#### 3.2.2 Comparison of the TBEV strains reproduction during mono- and mixed infection of ticks

The general appearance of the dynamics of the strains LK-Eu and Karl-Sib RNA copies accumulation during mixed infection is similar to that during mono-infection: an increase in the number of copies up to 3-7 days p.i. and then reaching a plateau with further insignificant fluctuations (**Supplementary Figures S4**). However, in almost all experimental groups, the RNA copy number of both strains in ticks was significantly higher during mono-infection than during mixed infection (**Supplementary Figures S4**), with the exception of the group ‘strain LK-Eu−*I. persulcatus* from Karelia’ (**Supplementary Figure S4A**). This may be due to both a lower infection dose with the strains during mixed infection and the limited resources of the tick’s body and the competition of TBEV strains for them.

## 4 Discussion

Circulation of TBEV in a natural focus is inextricably linked with ixodid ticks, which are not only vectors, but also reservoirs of the virus. The mechanisms underlying the establishment and maintenance of persistent viral infection in ticks are not completely understood since no direct experiments have been made, but apparently both host and viral factors are involved. As was shown in experiments with mosquitoes and their cell lines, among viral factors can be the presence of defective interfering particles (DIs), one of the major self-controlling mechanisms for viral replication, and among the host factors are RNAi and the innate immune responses that regulate but do not eliminate viral infections (Salas-Benito and De-Nova-Ocampo, 2015). In addition, it was shown that the 3’ UTRs of LGTV and TBEV express subgenomic flavivirus RNAs (sfRNAs), which are a counter defense against the tick RNAi system (Schnettler et al., 2014), and another way to maintain balance between vectors and viruses (Asgari, 2014).

Currently, changes in the ranges and abundance of ixodid ticks are occurring, which favor the circulation of TBEV in a non-specific species of ticks (Jääskeläinen et al., 2006, Jääskeläinen et al., 2011; Katargina et al., 2013). Many factors can influence the success of TBEV adaptation to a new vector species: the level of virus reproduction in the vector; success of overcoming intestinal and organ barriers and forming a persistent infection in the vector; the success of transovarial and trans-stadial transmission in ticks, the formation of viremia in the vertebrate host, etc. In the present work, we focused only on a detailed study of the reproduction level of the main TBEV subtypes in different tick species.

The reproduction and transmission of TBEV can be affected by the biological and physiological characteristics of ixodid ticks. For example, it has been shown that the TBEV reproduction level can be influenced by ticks feeding (Belova et al., 2012, Belova et al., 2018), diapause (Mishaeva and Erofeeva, 1979) and ticks’ physiological age (Mishaeva and Votyakov, 1978; Razumova and Alekseev, 1991). However, there is very little data on the interactions between TBEV subtypes and specific and non-specific tick species. There is evidence of a higher level of TBEV reproduction in the cell culture of ticks of a specific species (*I. ricinus* cell lines – TBEV-Eu) (Růžek et al., 2008a), higher efficiency of TBEV transmission to a specific tick species “*I. ricinus* – TBEV-Eu” during co-feeding (Khasnatinov et al., 2016), and in case of the second trans-stadial transmission in specific pairs “*I. ricinus* – TBEV-Eu” and “*I. persulcatus* – TBEV-Sib” (Belova et al., 2023).

Comparatively recently, data began to appear on the biological, physiological and genetic differences between ticks from different populations of the same species (Noureddine et al., 2011; Dinnis et al., 2014; Gilbert et al., 2014; Arsnoe et al., 2015). The possibility of specific adaptation between a tick population and the corresponding TBEV isolate was demonstrated in a study on nymphs using artificial feeding (Germany) (Liebig et al., 2021). However, no special studies have been conducted to date on the influence of different populations of ticks of the same species on the properties of different TBEV subtypes.

In our study, we used two populations of *I. ricinus, I. persulcatus* and *D. reticulatus* ticks collected in remote areas. The TBEV strains were selected in such a way that their places of isolation were more “close” to the one of the *I. ricinus* and *I. persulcatus* tick populations. Specific interaction was expected in the pairs “LK-Eu − *I. ricinus*, Curonian Spit”, “Karl-Sib − *I. persulcatus*, Karelia”, and “DV-FE − *I. persulcatus*, Tuva”. Despite the fact that we used strains of different TBEV subtypes in our work, we cannot extrapolate the results to virus subtypes until similar experiments are conducted with some other representatives of TBEV subtypes.

As a result of the work, we obtained data that *I. ricinus, I. persulcatus* and *D. reticulatus* effectively support the reproduction of TBEV regardless of the strain. Interpopulation differences in the maintenance of TBEV reproduction were noted only in *I. persulcatus*, where the reproduction level of all three TBEV strains was lower in ticks from Karelia than from Tuva. This may be due to the physiological state of ticks from Karelia, since they were characterized by the highest mortality in the experiment.

Despite minor differences in the level of virus reproduction in ticks, we found changes in the infectivity of TBEV strains for mammalian cell culture during reproduction in different species of ticks. The lowest infectivity for mammalian cells was observed for the strain Karl-Sib, regardless of the tick species, when compared with other strains, and the highest infectivity was noted for the LK-Eu strain. Association of this change with a specific tick species was observed only for DV-FE, for which the highest infectivity was registered in *I. persulcatus.*

The lowest infectivity of the Karl-Sib was due to the fact that this strain had a high percentage of samples that did not have plaques on PEK cells, but with fairly high RNA copy numbers. This fact may be due to the emergence of variants that are not infectious for mammalian cell cultures or to the accumulation of DIs. Reduced detection of infectious particles of the strain Karl-Sib was observed in all experimental groups of ticks and at different time after infection. However, this phenomenon was most pronounced in *I. persulcatus* from Karelia, the most specific tick population for this TBEV strain. Probably, this is one way of virus adaptation to persistence in the tick’s body. Changes in infectivity may indicate the probability of the emergence of a new TBEV variants, however, only studying the pathogenicity of the virus and viremia level in animal models can give us reliable information about changes in the properties of the virus in relation to mammals.

In addition to the dynamics of accumulation of RNA copies and infectious particles of TBEV strains, the plaque phenotype on the PEK cell culture, which is an important property of the viral population, was analyzed in variants after persistent mono-infection for 39-79 days. Thus, a change in the plaque phenotype for TBEV variants was described after a change in the reproduction system (Romanova et al., 2007; Růžek et al., 2008b; Belova et al., 2017). In our studies, each strain had a different phenotypic characteristic, which gradually changed after replication in ticks: all strains were characterized by the appearance of phenotype variants with smaller plaques than in the original. For the strain Karl-Sib, the appearance of large plaques was noted during reproduction in *D. reticulatus*. The smallest phenotypic changes were characteristic to the strain LK-Eu during reproduction in a specific population of *I. ricinus* ticks from the Curonian Spit.

Characterizing the TBEV variants obtained after persistence in ticks, we observed changes in the plaque phenotype in PEK cell culture and infectivity for mammals cells with similar dynamics of RNA copies accumulation. The data obtained allow us to conclude that during TBEV adaptation to the vector, the properties of the viral population are affected.

For a more detailed study of the relative adaptability of the TBEV-Eu and TBEV-Sib to tick species, a mixed infection of different tick species with strains of these subtypes was carried out. In case of mixed infection, the ability of virus strains to compete during reproduction in the same system is assessed. It was supposed that these interaction will result in heterotypic viral interference, since these viruses have similar mechanisms of translation/replication. The mechanisms of viral interference can involve different levels of the viral replicative cycle, such as binding, entry, replication, and morphogenesis. Various factors as presence of DIs, RNAi response, competition for cellular replication factors and the innate immune response can be responsible for the viral interference (Salas-Benito and De-Nova-Ocampo, 2015). However, no experimental studies on the relationships between different TBEV subtypes in ixodid ticks have been conducted to date. Our experiments showed that LK-Eu and Karl-Sib strains had approximately the same ability to reproduce during mixed infections in specific and non-specific ticks. In *Ixodes* ticks from European populations (*I. persulcatus* − Karelia, *I. ricinus* − Curonian Spit), in which both TBEV subtypes can be found in nature, the reproduction level of both strains was similar throughout the observation period, and their ratio in the tick was almost the same or with a slight dominance of LK-Eu. However, in *I. persulcatus* ticks from Tuva and *I. ricinus* ticks from the Voronezh region, the Karl-Sib strain dominated throughout the observation period. For ticks from Tuva, this observation is logical, since the Siberian TBEV subtype dominates in the Republic, and this subtype has probably formed a specific connection with ticks of the local population. The Voronezh region is not endemic for TBEV, but in the nearest TBEV-endemic territory, the Siberian subtype is dominant. Perhaps, relationships between strains only in these two tick populations can be attributed to heterotypic viral interference, but currently we cannot say anything about its mechanisms.

In *D. reticulatus* populations, which are not specific vectors of TBEV, the reproduction dynamics of LK-Eu and Karl-Sib were similar and the ratio of these strains in the tick starting from 24 h after infection was almost the same. The obtained results indicate the existence of adaptation of European and Siberian strains of TBEV to local populations of *I. persulcatus* and *I. ricinus* ticks.

Thus, we demonstrated that the level of virus reproduction is not the primary factor that determines the adaptation of TBEV to a new tick species. The nature of changes in TBEV infectivity during adaptation to different tick species depends on the virus strain and the species of ticks.

## Data Availability

The original contributions presented in the study are included in the article/**Supplementary Material**. Further inquiries can be directed to the corresponding author.
