# Supplementary material for: Tick-borne encephalitis virus subtypes: mono- and mixed infection in specific and non-specific ticks
Source: Front Cell Infect Microbiol. 2025 Mar 26;15:1568449. doi: 10.3389/fcimb.2025.1568449 (PMC11979232; doi:10.3389/fcimb.2025.1568449)
Supplement: Supplementary file 1 [file DataSheet1.pdf]

Supplementary Material

Table S1. Specific primers used in the study

Target virus	Primer name	Target genome region	Oligonucleotide sequence	Where was used
TBEV, all strains	Pow-TBE-3	3`NTR	5′-AGCGGGTGTTTTTCCGAGTC-3′	RT; standards preparation
	R-TBE		5′-ACAC ATCA CCTC CTTG TCAGACT-3′	qPCR
	F-TBE		5′-GGGCGGTTCTTGTTCTCC-3′	qPCR
	TBE-probe		5′-(FAM*)-TGAGCCAC CATC ACCCAGACACA-(BHQ1*)-3′	qPCR
	BHT7kgg23	NS5	5′-ATGACTGGATCCTAATACGACTCACTATAGGTACTTTCTGAATGACATGGC-3′	standards preparation
Poliovirus	PVR1	3Dpol	5′-CGAACGTGATCCTGAGTGTT-3′	RT, qPCR, qPCR-Subtypes
	PVL1		5’-GGCAGACGAGAAATACCCAT-3’	qPCR, qPCR-subtypes
	PVP		5’-(R6G)-TTGATTCATGAATTTCCTTCATTGGCA-(BHQ1)-3’	qPCR, qPCR-subtypes
TBEV, strain LK-138	TBE-E1	C	5’-CATGCCGTAGCTGGCACCGCGAGAAA-3’	qPCR-subtypes
	TBE-E2	K	5’-TCGACCAACGACCAGTAATGAGACAAC-3’	RT, qPCR-subtypes
	TBE-E4-new	C	5’-(FAM)-CAGAGGGACTGAGTTCCAGAACGCCT-(BHQ1)-3’	qPCR-subtypes
	BHT7PowF	5`NTR	ATGACTGGATCCTAATACGACTCACTATAGGAGATTTTCTTGCACGTGT	standards preparation
	1095r	E	5’-GMGTCAAGCCACACATCC-3`	standards preparation
TBEV, strain Karl08-T3522	TBE-31.1-new	NS1	5’-ACCAGTGACTGRACTCTTTRACGAGTAC-3’	RT, qPCR-subtypes
	TBE-29.1-new	NS1	5’-TTYYTGGAYTTYAGACAGGAASCAACACA-3’	qPCR-subtypes
	TBE-Karl	NS1	5’-(ROX)-TGACACTGGAACTTACATAGTTGAGTTAT-(BHQ2)-3’	qPCR-subtypes

	BHT7_Kgg16	E	5`-ATGACTGGATCCTAATACGACTCACTATAGGAGGGGAGCAGCATTGGAAG-3`	standards preparation
	Kgg30	NS1	5`-TGGTGCTCCTCACAGAAGC-3`	standards preparation

qPCR- Quantitative Real-Time PCR, RT- Reverse Transcription, qPCR-subtypes - Quantitative Real-Time PCR for different subtypes of TBEV infection

Table S2. Results of the strains LK-Eu and Karl-Sib detection in various combinations using RT-PCR system.

Sample, log(GCP/2 μL)	strain LK-Eu		strain Karl-Sib	
	Cq	log(GCP/2 μL)	Cq	log(GCP/2 μL)
LK-Eu, 4.5	27.01	4.5	-	-
LK-Eu, 8	15.02	8.0	-	-
Karl-Sib, 4.5	-	-	25.72	4.2
Karl-Sib, 6.7	-	-	16.64	6.7
LK-Eu/Karl-Sib, 7.5/6.7	17.75	7.2	16.72	6.7
LK-Eu/Karl-Sib, 7.5/4.5	17.07	7.4	26.25	4.0
LK-Eu/Karl-Sib, 4.5/6.7	27.46	4.4	18.01	6.3
LK-Eu/Karl-Sib, 4.5/4.5	27.61	4.3	24.23	4.6

GCP – genome copies number

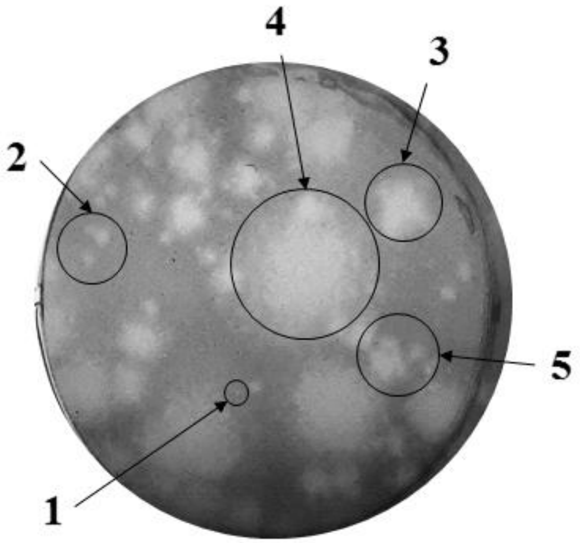

Phenotype	Characteristic
1	small pinpoint
2	d = 1 – 1,5 mm
3	d = 2 – 4 mm
4	d > 4 mm
5	various sizes with indistinct, irregular star-shaped contour

Figure S1. The TBEV plaque phenotypes in PEK cell culture on the seventh day of infection.

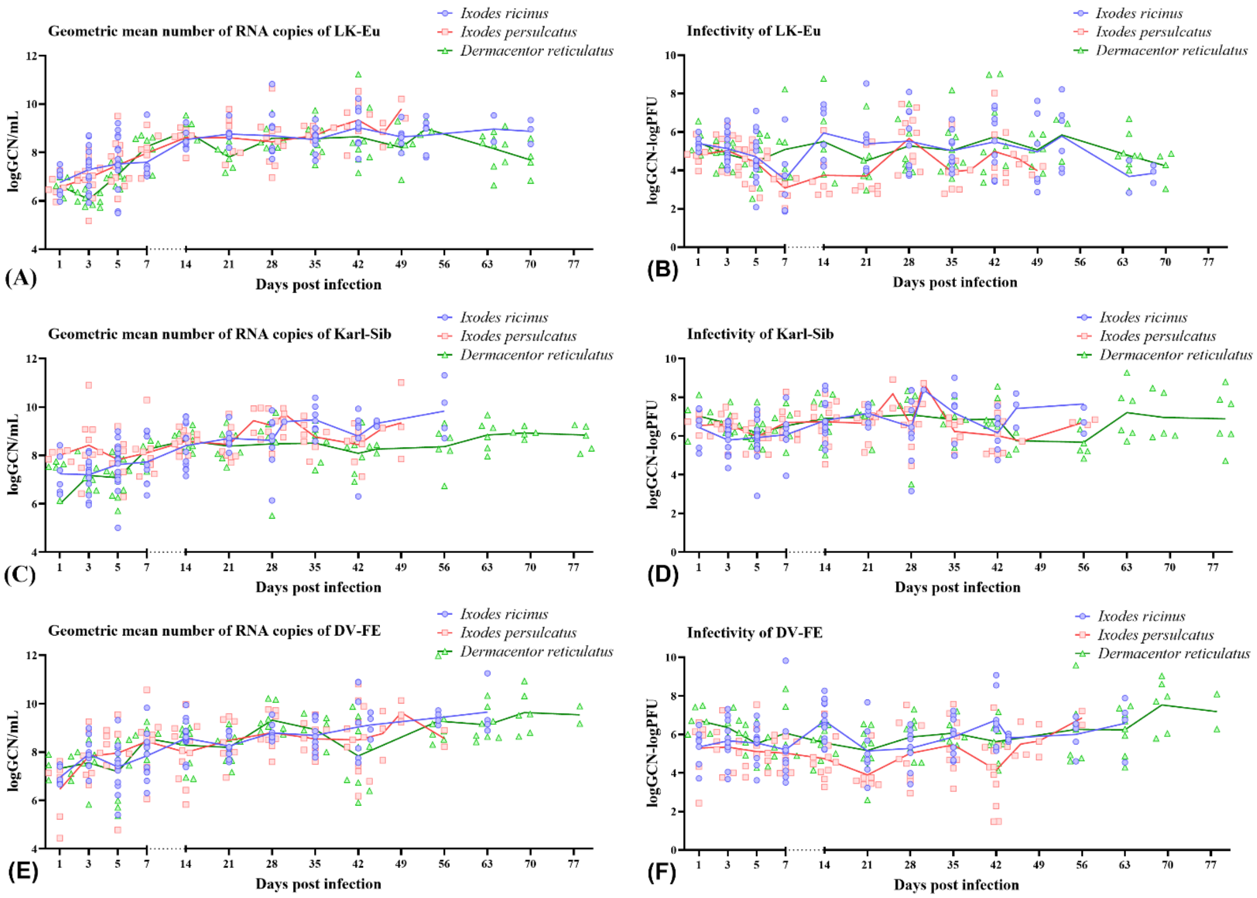

Figure S2. Dynamic of the RNA copies number (A, C, E) and infectivity (B, D, F) of strains LK-Eu (A,B), Karl-Sib (C,D), DV-FE (E,F) TBEV in ticks *Ixodes ricinus* (blue), *I. persulcatus* (red), *Dermacentor reticulatus* (green). GCN/mL – genome copies number in mL of the 0.17% tick suspension, infectivity, expressed as decimal logarithm of the ratio of RNA copies to the titer of infectious virus (logGCN - logPFU).

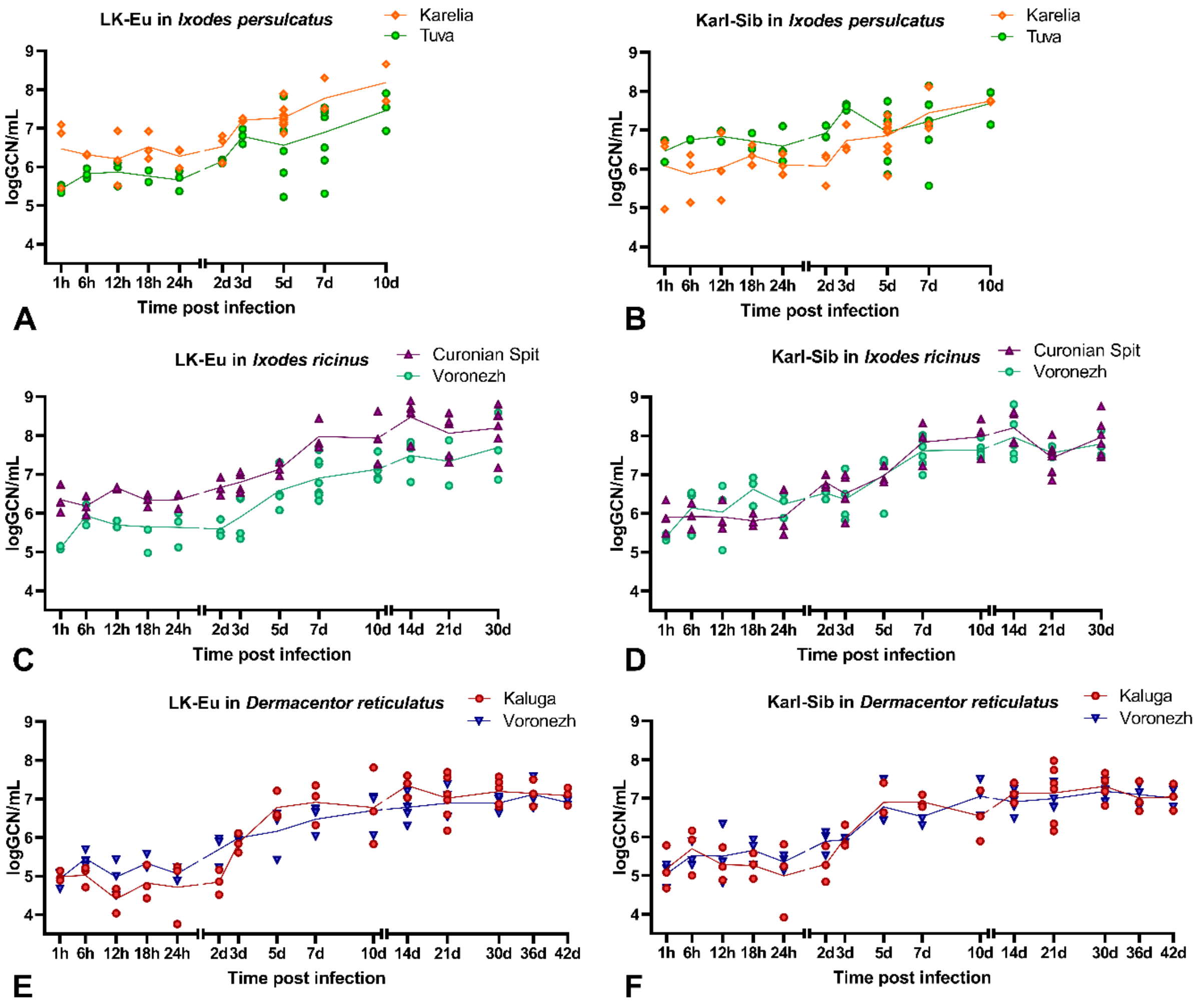

Figure S3. Dynamic of the RNA copies number of the strains LK-Eu (A, C, E) and Karl-Sib (B, D, F) during mixed infection of two populations of *I. persulcatus* (A, B), *I. ricinus* (C, D), and *D. reticulatus* (E, F). GCN/mL – genome copies number in mL of the 0.17% tick suspension.

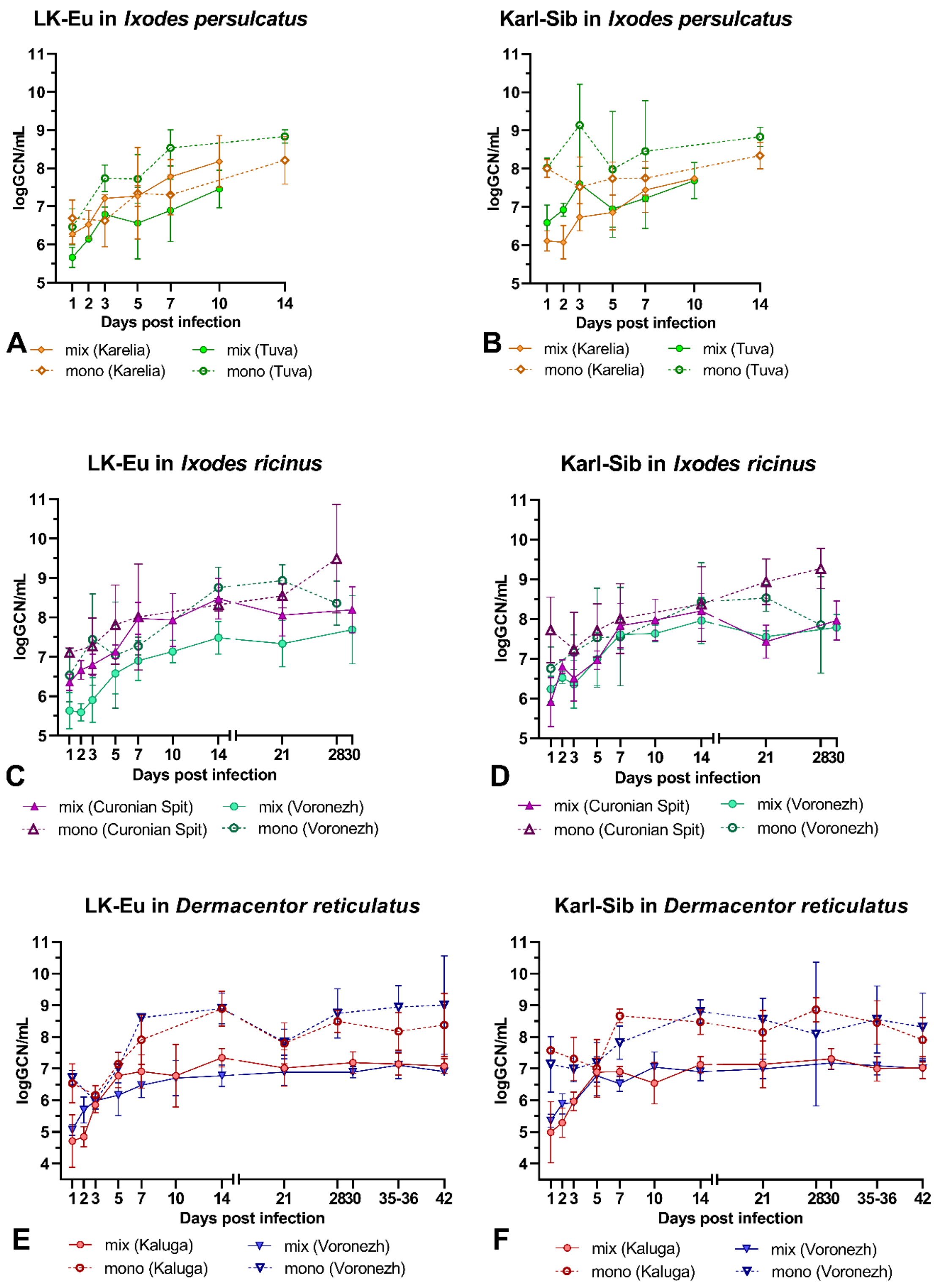

Figure S4. Comparison of the dynamics of the RNA copies number of the strains LK-Eu (A, C, E) and Karl-Sib (B, D, F) during mono- (dashed line) and mixed (continuous line) infection of two populations of *I. persulcatus* (A, B), *I. ricinus* (C, D), and *D. reticulatus* (E, F). GCN/mL – genome copies number in mL of the 0.17% tick suspension.
